# Mahuang Fuzi Xixin decoction ameliorates allergic rhinitis and repairs the airway epithelial barrier by modulating the lung microbiota dysbiosis

**DOI:** 10.3389/fmicb.2023.1206454

**Published:** 2023-08-14

**Authors:** Xiaohan Wei, Mengze Ding, Xiao Liang, Baoping Zhang, Xiaomei Tan, Zezhong Zheng

**Affiliations:** ^1^School of Traditional Chinese Medicine, Southern Medical University, Guangzhou, China; ^2^Guangdong Provincial Key Laboratory of Chinese Medicine Pharmaceutics, Southern Medical University, Guangzhou, China; ^3^School of Pharmaceutical Sciences, Guilin Medical University, Guilin, China; ^4^South China Agricultural University College of Veterinary Medicine, Guangzhou, China

**Keywords:** allergic rhinitis, lung microbiota, metabolism, airway barrier, *Pseudomonas aeruginosa*

## Abstract

**Background:**

Allergic rhinitis (AR) is a common disorder, that burdens general well-being. Although the lung is connected to the upper respiratory tract, which is rich in microorganisms, no studies have reported the relationship between lung microbiota and AR. Mahuang Fuzi Xixin decoction (MFXD) is a traditional Chinese medicine (TCM) formula that is widely used to treat AR in the clinic but its underlying mechanism remains unclear.

**Hypothesis:**

We hypothesized that lung microbiota is associated with the pathogenesis of AR, and MFXD can improve AR by regulating microbiota dysbiosis.

**Methods:**

The ovalbumin-induced mouse AR model was used to evaluate the therapeutic effect of MFXD on AR. Then 16S rDNA amplicon sequencing, untargeted metabolomics, and other molecular biology technology were used to clarify the effects of MFXD on lung microbes dysbiosis and AR progression. Further, the human nasal epithelial cell line (HNEpCs) was used to evaluate the protective effect of MFXD on epithelial barrier damage caused by specific pathogens.

**Results:**

MFXD decreased plasma histamine and IgE levels, ameliorated pathological damage, and diminished the expression of tight junction proteins (ZO-1 and occludin) in lung and nasal tissues. MFXD altered AR-induced microbiota dysbiosis in the lungs and also plasma metabolites. Oral administration of MFXD altered microbiota dysbiosis in lung and AR-associated metabolic disorders. The dominant bacteria in the lungs of AR mice damaged the airway barrier, and MFXD reversed this change.

**Conclusion:**

This study revealed the correlation between the lung microbiota and AR in the mice model. We confirmed that lung microbiota plays a vital role in AR and that MFXD reduced damage to the epithelial barrier of the lungs and nasal mucosa by regulating lung microbiota and plasma metabolism imbalances. Our research provides a reference for the effect of lung microbiota on AR and provides a new idea for the treatment of AR.

## 1. Introduction

Allergic rhinitis (AR) is a prevalent disorder among humans, especially in adolescents, burdening general well-being, and its incidence continued to increase in recent years (Zhang et al., 2021). AR is closely related to asthma, approximately 40% of patients with AR report asthma symptoms, and approximately 80% of patients with asthma also have symptomatic AR, which indicates that the upper and lower airways have similar disease associations and immunopathological mechanisms (Samitas et al., 2018). Patients with AR who do not have asthma still show inflammation of the lower airway (Togias, 2001). The epithelium acts as a barrier against external allergens and stimuli and participates in appropriate immune responses. Damage to the tight junction of the epithelial barrier is a part of the underlying pathology of asthma and AR (Steelant et al., 2018).

The gut microbiome is a complex ecosystem that plays an important role in disease development and immune homeostasis. The pathogenic microbiome may aggravate disease development by causing mucosal barrier dysfunction (Shi et al., 2017). However, healthy lungs are considered sterile before. The development of sequencing technology has resulted in increased publications on the lung microbiome. Microbiota enters the lungs mainly through the nasopharynx and oropharynx, and pathogenic bacteria may colonize the paranasal sinuses in the early stage and migrate to the lower airways (Hansen et al., 2012; Barcik et al., 2020). Lung microbiota may be closely related to the pathogenesis of airway diseases. Moreover, the microbiota in the lungs is associated with changes in the immune response and the development of lung disease (Whiteside et al., 2021). Currently, the relationship between lung microbiota and AR development is still inconclusive. Therefore, it is important to explore the changes in the lung microbiota during AR.

Mahuang Fuzi Xixin decoction (MFXD) is a traditional Chinese medicine (TCM) formula, it was first recorded in “Treatise on Febrile Diseases” (Shang Han Lun) compiled by Zhongjing Zhang of the Han Dynast comprising three herbs: MaHuang (the stem of *Ephedra sinica* Stapf.), Fuzi (the root of *Aconitum carmichaeli* Debx.), and Xixin (the root of *Asarum sieboldii* Miq.). It is traditionally used to treat colds, asthma, and AR. MFXD regulated the Th1/Th2 and Th17/Treg cell balance in rat AR models (Liang et al., 2020). However, whether MFXD affects the lung microbiota and the correlation between lung microbiota and AR have not been studied. In this study, we used 16S rDNA amplicon sequencing, untargeted metabolomics, and molecular biology technology to clarify the relationship between lung microbiota and AR, and the effects of MFXD on plasma metabolites and potential pathogens. We proved that MFXD can reverse the changes in the lung microbiota of mice with AR. It also reduced damage to the epithelial barrier of the lung and nasal mucosa by regulating the dysbiosis of lung microbes and plasma metabolites. This provides a new perspective for research on AR diseases and drugs.

## 2. Materials and methods

### 2.1. Reagents

MaHuang was purchased from Sinopharm Group Feng Liao Xing Medicinal Materials Co., Ltd. (Foshan, China); Fuzi was purchased from Guangzhou Baiyunshan Pharmaceutical Group Co., Ltd. (China); Xixin was purchased from Anhui Tonghuatang Medicinal Materials Co., Ltd. (China); Ovalbumin (OVA) and FITC-FD4 were obtained from Sigma-Aldrich (St Louis, MO, United States) and aluminum hydroxide was purchased from Damao Chemical Reagent Factory (Tianjin, China); Dexamethasone (Dex) was purchased from Aladdin (Shanghai, China); CCK-8 was purchased from Glpbio (Montclair, CA, United States); The primary antibodies against ZO-1 and Occludin were purchased from Proteintech (Rosemont, IL, United States);

### 2.2. Methods

#### 2.2.1. Preparation of MFXD

We prepared MFXD according to clinical application: Mahuang, Fuzi, and Xixin were weighed in a ratio of 2:3:1. The Mahuang was soaked in 30 times water for 30 min and boiled for 20 min. Subsequently, Fuzi and Xixin were added and the solution was decocted for 90 min. After filtration, MFXD was concentrated at 1.1 g/mL for animal administration. Part of the concentrate was frozen into Mahuang Fuzi Xixin decoction lyophilized powder (MFXD-LP) for cell and bacterial experiments.

#### 2.2.2. Animal study

##### 2.2.2.1. Animals

Male BALB/c mice (SPF grade, 18–22 g) were provided by the Laboratory Animal Center of the Southern Medical University and reared in specific pathogen-free (SPF) conditions. All animal experiments have passed the resolution of the Animal Ethics Committee of Southern Medical University (Resolution No. SMUL2019087; Date of Resolution 2019.5.10).

##### 2.2.2.2. Animal treatment

According to similar research (Thi et al., 2019), thirty mice were randomly divided into 5 groups: naïve (untreated group), OVA, Dex (2.5 mg/kg), MFXD low dose (1.375 g/kg, MFXD-L), and MFXD high dose (MFXD-H, 2.75 g/kg). The doses of Dex and MFXD-H (2.75 g/kg) were converted from human oral dose (Liang et al., 2020). Except for the naïve group, 200 μL of normal saline containing 1 mg aluminum hydroxide, and 50 μg OVA were injected intraperitoneally on days 1, 7, and 14 for basic sensitization. On days 15–27, Dex (2.5 mg/kg) or MFXD (1.375 or 2.75 g/kg) were orally administered to mice once a day. On days 21–27, mice were stimulated intranasally with 20 μL of OVA solution. On day 27, the number of mice presenting with itching and sneezing in each group within 15 min was evaluated by blinded observers. The mice were sacrificed on day 28, and blood, head, lung, and fresh fecal samples were collected.

##### 2.2.2.3. Enzyme-linked immunosorbent assay

Blood was harvested in a tube containing EDTA and centrifuged (1,000 × g, 4°C, 10 min) to separate the plasma. Plasma was used to determine histamine and IgE levels using an enzyme-linked immunosorbent assay (ELISA) kit (Jiangsu Meimian Industry Co., Ltd. Yancheng, China).

##### 2.2.2.4. Hematoxylin and eosin and immunohistochemistry staining

The heads and the middle lobe of the lungs of the mice were fixed with 4% paraformaldehyde (heads were decalcified in ethylenediaminetetraacetic acid solution for another 6 days) and then embedded in paraffin. The specimens were then cut into 5-mm-thick slices and stained with Hematoxylin and eosin (H&E). Immunohistochemistry (IHC) staining was used to evaluate the expression of tight junction proteins in the nasal mucosa and lungs. ZO-1 (1:1000) and occludin (1:1000) were used as primary antibodies and labeled specifically using the DAB substrate kit (Beyotime, Shanghai, China).

##### 2.2.2.5. 16S rDNA amplicon sequencing

Lung tissues were stored at −80°C and thawed during testing. Total DNA was extracted using the EZNA Stool DNA Kit, according to the manufacturer’s instructions. Polymerase chain reaction (PCR) amplification of the 16S rDNA V3–V4 region was performed. The primers used were 341F (CCTACGGGNGGCWGCAG) and 806R (GGACTACHVGGGTATCTAAT). 30 ng DNA samples and fusion primers were used for the PCR system and sequenced on an Illumina MiSeq platform. Raw reads were filtered and added tags by Fast Length Adjustment of Short reads program version 1.2.11. The tags were then clustered into operational taxonomic units (OTU) by UPARSE software with a cutoff value of 97%, and UCHIME (version 4.2.41) was used to compare chimera sequences to the Gold database. Following this, we use Ribosomal Database Project (RDP) Classifier v.2.2 to classify OTU sequences and train on the Greengenes database. All Tags were compared to OTUs using USEARCH_global to obtain the OTU abundance statistics of each sample.

##### 2.2.2.6. Plasma metabolomic analysis

Plasma samples (100 μL) were extracted with 300 μL of precooled methanol and acetonitrile (2:1, v/v), followed by the addition of internal standards mix 1 (IS1) and internal standards mix 2 (IS2) to control the quality of sample preparation. After Vortex for 1 min, the samples were incubated at −20°C for 2 h, then centrifuged (20 min, 1,800 × g) and the supernatants were transferred to vacuum freeze-drying. 150 μL of 50% methanol was added to resuspend the metabolites, centrifuged (30 min, 1800 × g), and the supernatants were transferred to autosampler vials for liquid chromatography-mass spectrometry (LC-MS) analysis. Ultra-high performance liquid chromatography (UPLC-MS) systems consisted of Ultra high-performance liquid phase (Waters 2D UPLC, Waters, Milford, MA, United States) and high-resolution mass spectrometry (Q Exactive HF, Thermo Fisher Scientific, Waltham, MA, United States). The same volume of each sample was pooled and prepared as quality control (QC) samples to assess the reproducibility of the whole LC-MS analysis. The compounds were identified using an online library including METLIN^1^ and HMDB,^2^ which were matched with mass spectrum (MS) and secondary mass spectrum (MS/MS) data. The data were then processed by multi-variate statistical analysis including principal component analysis (PCA) and volcano plot analysis by R package. Kyoto Encyclopedia of Genes and Genomes (KEGG)-based metabolic enrichment analysis was constructed using PATHVIEW.^3^

##### 2.2.2.7. Correlation analysis

The Spearman correlation coefficients between the lung microbiota (genus) and the plasma metabolites were calculated and visualized using R (version 3.4.1).

#### 2.2.3. Bacterial and cell validation

##### 2.2.3.1. Preparation and quantitation of *Pseudomonas aeruginosa* exoprotein

*Pseudomonas aeruginosa* (ATCC27853) was obtained from Huankai Biological Co., Ltd. (Guangzhou, China). The bacteria were cultivated on *Pseudomonas* agar base medium (Huankai Biological) at 37°C with agitation at 1.1 × g in a bacteriological incubator. After the bacteria were cultured for 12 h, the *P. aeruginosa* exoprotein (PAE) was collected. The medium was centrifuged for 10 min at 7000 × g and filtered through 0.22 μm filters. The supernatant was concentrated using a centrifugal vacuum concentrator (EYELA, Tokyo, Japan), and quantified using the BCA protein assay kit (Keygen Biotech, Jiangsu, China).

##### 2.2.3.2. Growth curve of *Pseudomonas aeruginosa*

*P. aeruginosa* (1%) were inoculated in LB broth containing 0.625, 1.25, 2.5, 5 mg/mL MFXD lyophilized powder, the OD_600_ values were measured every 1 h for 14 h. An uninoculated drug-containing medium was used as the control, and growth curves were plotted.

##### 2.2.3.3. Cell culture and cell viability assay

HNEpCs were human nasal epithelial cell lines obtained from Jennio Biotech Co., Ltd. (Guangzhou, China), and cultured in minimum essential media (Gibco, Grand Island, NY, United States) and 10% fetal bovine plasma (ExCell Bio, Shanghai, China) with 1% penicillin-streptomycin solution and placed in a 37°C, 5% CO_2_ incubator. The cells were seeded in 96-well plates (5,000 *per* well). After adherence, the cells were incubated with MFXD-LP 12.5, 25, and 50 μg/mL for 24 h. Cell viability was then determined using the CCK-8 assay, at 450 nm with Multiscan Spectrum (Thermo Scientific).

##### 2.2.3.4. Trans epithelial electrical resistance (TEER) analysis and FITC-FD4 permeability assay,

HNEpCs were seeded on transwell inserts (0.4-μm polyester membrane, diameter 24 mm) in 6-well plates (Corning, MA, United States) at a density of 1 × 10^5^ cells *per* transwell. The cultural medium was replaced every day. When the cells grew to complete confluence, trans epithelial electrical resistance (TEER) was measured by Millicell^®^ ERS-2 voltmeter (Merck Millipore, Darmstadt, Germany). A tight monolayer was considered to be formed only if the resistance is greater than 300 Ω cm^2^. The cells were pretreated with 12.5, 25, and 50 μg/mL MFXD-LP for 24 h and then treated with 100 μg/mL PAE, in the presence or absence of 12.5, 25, and 50 μg/mL MFXD for another 4 h. Subsequently, the TEER in the different wells was measured and normalized to the average measured value before adding PAE and after 4 h.

FITC-FD4 was used to assess the paracellular permeability. Briefly, FITC-FD4 (100 μg/mL) was added to the upper chambers and incubated for 2 h to assess paracellular permeability. During that, 200 μL of the samples were collected from the bottom chamber at 30, 60, and 120 min and evaluated using a fluorescence microplate reader (BioTek, Santa Clara, CA, United States).

##### 2.2.3.5. Immunofluorescence analysis

HNEpCs (2 × 10^5^ cells *per* well) were transferred to 6-well plates and treated with MFXD-LP at 12.5, 25, and 50 μg/mL in the presence or absence of PAE (100 μg/mL) for 24 h. After fixation in 4% paraformaldehyde for 15 min, the cells were incubated with primary antibodies against ZO-1 (1:1000) and occludin (1:1000) overnight at 4°C, followed by incubation with the secondary antibody, Alexa Fluor 488 or Alexa Flour^®^ 594 goat anti-rabbit (Beyotime, Jiangsu, China) for 2 h at room temperature. Cell nuclei were stained with DAPI (Beyotime) for 10 min. The stained cells were observed using a DMi8 microscope (Leica Microsystems, Wetzlar, Germany).

#### 2.2.4. Identification of ingredients in MFXD

MFXD was diluted to 50 mg/mL with 70% methanol. The sample was analyzed using UPLC-Orbitrap-HRMS (ThermoScientific, Sunnyvale, CA, United States) with an ACE Excel 3 C18 (100 mm × 2.1 mm, 2.1 μm) column (ACE, Aberdeen, United Kingdom). The mobile phase consisted of 0.1% formic acid (A) and acetonitrile (B) with an elution gradient: 0–15 min, 5%–12% B; 15–20 min, 12%–30% B; 20–30 min, 30%–95% B; 30–35, 95% B. The injection volume was 5 μL, the flow rate was 0.3 mL/min, and the electrospray ionization source was in positive and negative modes.

### 2.3. Statistical analysis

The level of significance between different groups was assessed by one-way analysis of variance (ANOVA) followed by Tukey’s test using GraphPad Prism 7. All data are displayed as mean ± SD, with a significance criterion of *p* < 0.05.

## 3. Results

### 3.1. MFXD alleviated itching, sneezing, and pathological symptoms in OVA-induced AR mice

**Figure 1:**
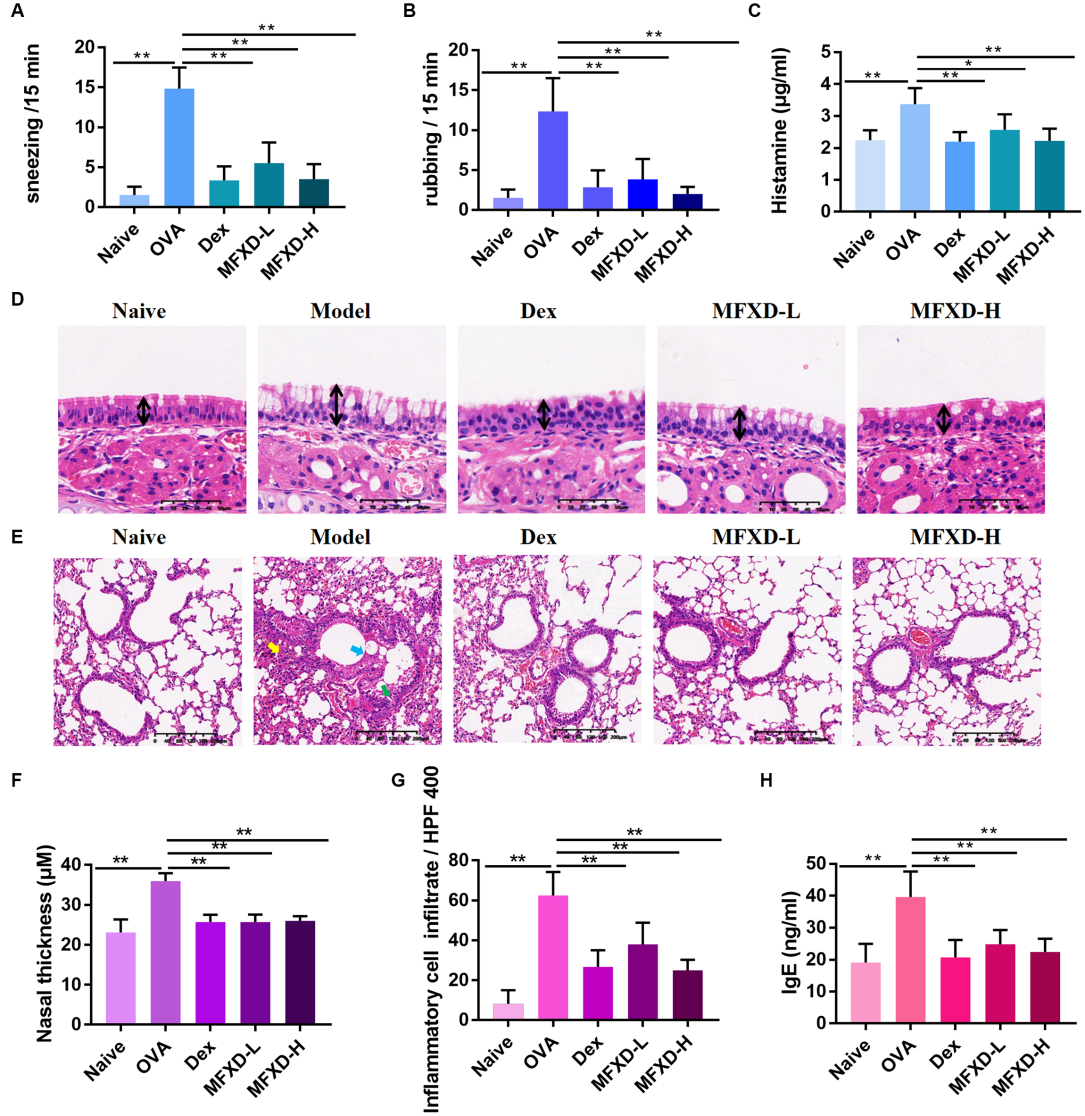
Mahuang Fuzi Xixin decoction (MFXD) alleviated **(A)** sneezing, **(B)** rubbing, **(D–G)** pathological symptoms, and attenuated excessive secretion of **(C)** histamine, and **(H)** IgE in ovalbumin-induced allergic rhinitis mice. ^*^p < 0.05, ^**^p < 0.01.

The OVA-induced AR mouse model showed clinical symptoms similar to those of AR, such as sneezing and nasal itching. OVA stimulation significantly increased the incidence of sneezing and rubbing (*p* < 0.01) in mice, which was significantly improved after treatment with MFXD (1.375 and 2.75 g/kg) and Dex (Figures 1A,B). The nasal mucosa (Figures 1D,F) of the mice in the OVA group was significantly thickened (*p* < 0.01) and goblet cells proliferated. Moreover, AR mice showed excessive bronchial mucus secretion (Figure 1E, blue arrow), bronchial mucosal damage (Figure 1E, green arrow), and peribronchial inflammatory infiltration (Figures 1E,G, yellow arrow). These symptoms were reduced by MFXD and Dex, and MFXD was more effective than Dex in reducing lung symptoms.

### 3.2. MFXD attenuated OVA-induced excessive secretion of inflammatory factors

We measured the contents of histamine and IgE in the plasma of mice from different groups. The plasma histamine of mice in the OVA group reached 3.373 ± 0.50 μg/mL, while IgE reached 39.63 ± 7.99 ng/mL, which were significantly down-regulated after oral administration of MFXD or Dex (Figures 1C,H).

### 3.3. MFXD restored nasal mucosal epithelium and lung barrier function in OVA-induced AR mice

**Figure 2:**
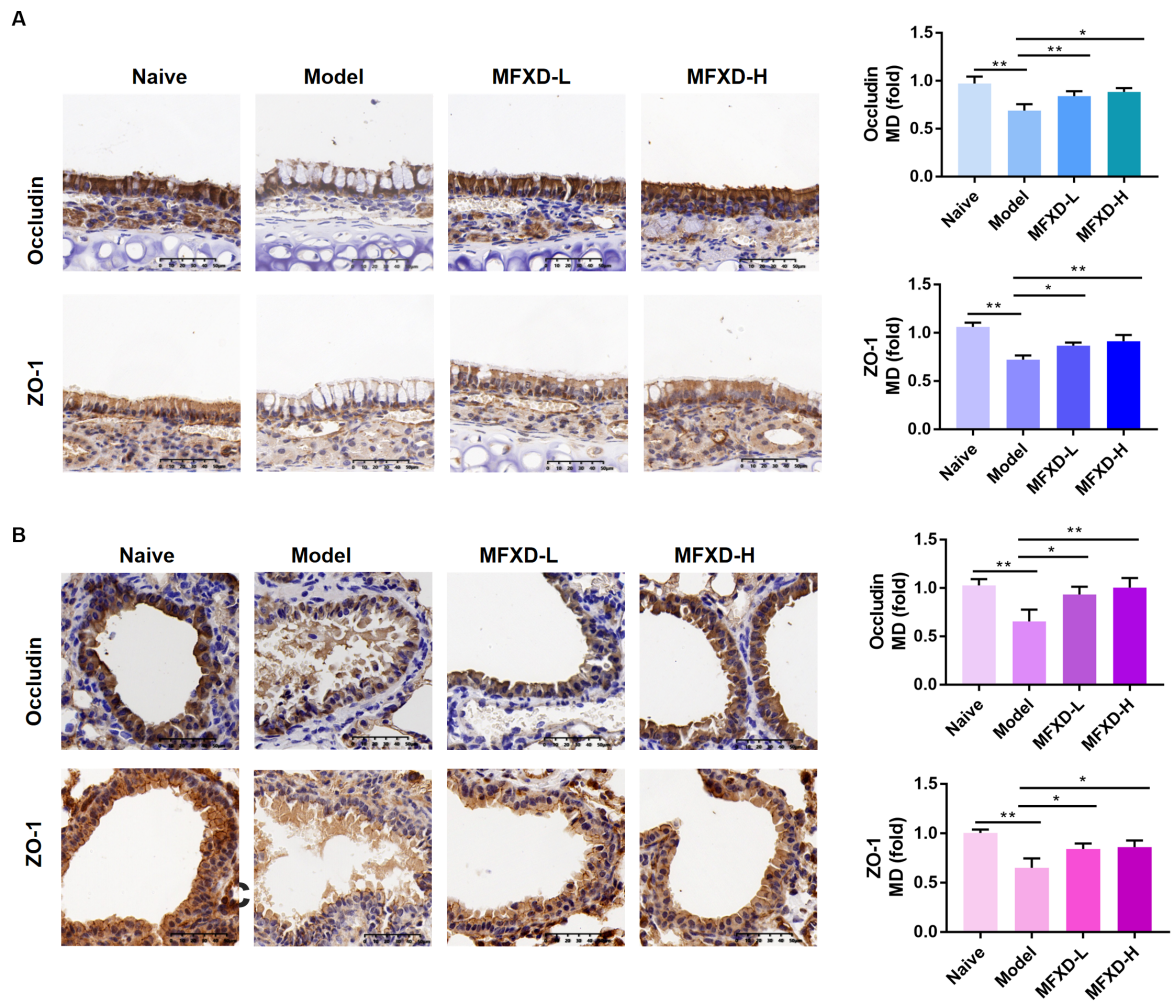
The expression of tight junction proteins (occludin and ZO-1 and) in **(A)** nasal mucosa and **(B)** bronchial epithelium of mice in different groups. ^*^p < 0.05, ^**^p < 0.01.

Immunohistochemistry was used to evaluate the expression of tight junction proteins (occludin and ZO-1) in the nasal mucosa and bronchi of mice in the different groups. As shown in Figure 2, the expression of ZO-1 and occludin were significantly (*p* < 0.01) decreased in the nasal mucosa and bronchi of mice in the OVA group, oral administration of MFXD (1.375 and 2.75 g/kg) markedly increased their expression. This indicates that MFXD can restore nasal mucosal epithelium and lung barrier function in OVA-induced AR mice.

### 3.4. MFXD altered the lung microbiota dysbiosis in OVA-induced AR mice

**Figure 3:**
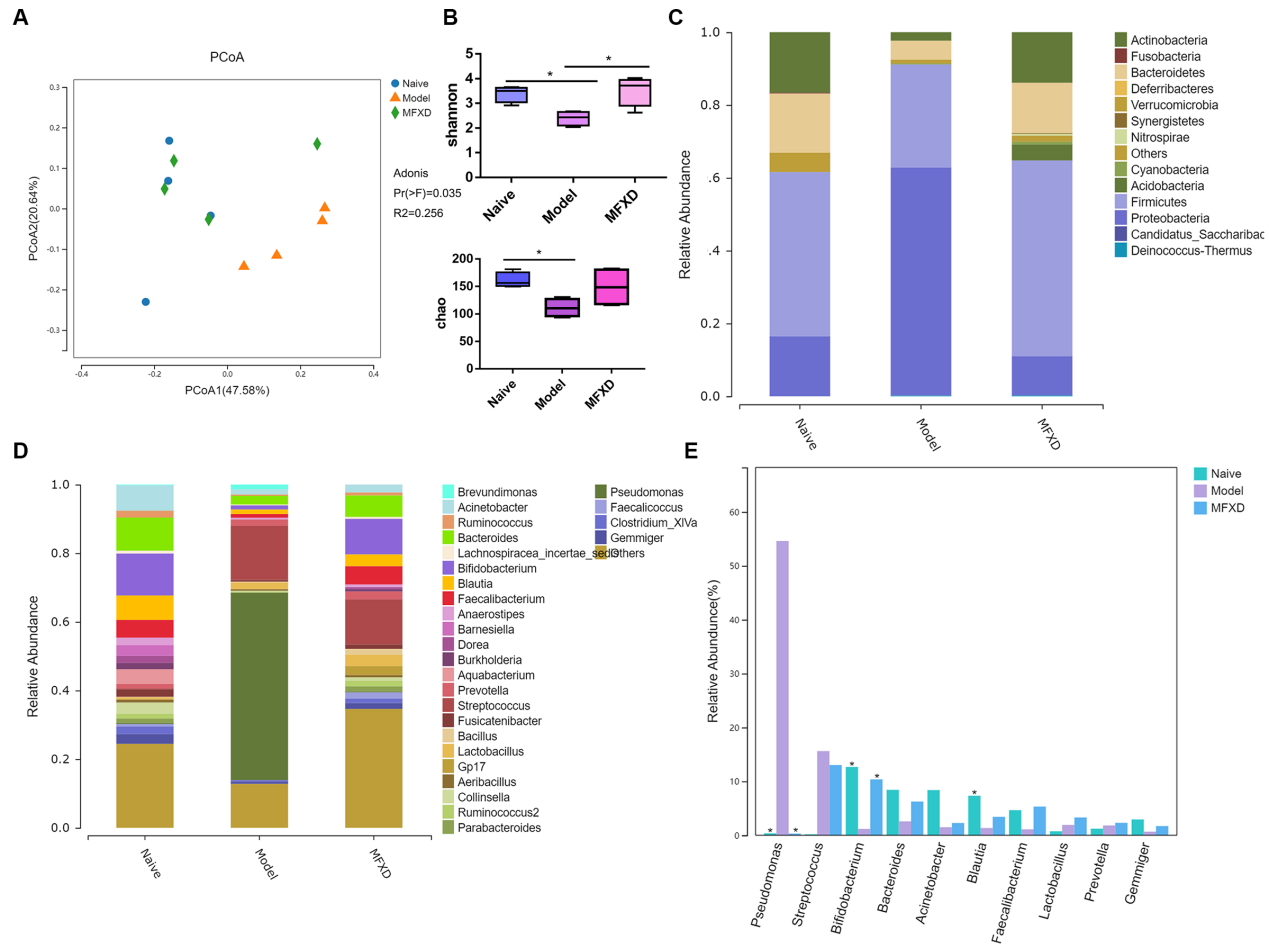
Mahuang Fuzi Xixin decoction (MFXD) altered the lung microbiota dysbiosis in ovalbumin-induced allergic rhinitis mice. **(A)** Principal coordinates analysis (PCoA) of lung microbiota based on the operational taxonomic unit (OTU) data. **(B)** Alpha diversity analysis (Shannon index and Chao index). **(C)** Community distribution of lung microbiota at the phylum level. **(D)** Community distribution of lung microbiota at the genus level. **(E)** Difference bar chart of top 10 species in relative abundance at the genus level. ^*^p < 0.05.

The principal coordinates analysis (PCoA) based on OTUs showed different trends between naïve and AR model groups in the lung; the MFXD group was different from the model group and showed similar trends to the naïve group (Figure 3A). The Shannon and Chao index in the lung microbiota of model group are significantly decreased (*p* < 0.05) compared with those of naïve group, suggesting variation of microbes in AR group were significantly altered, which were increased in MFXD group (Figure 3B).

At the phylum level, the AR model group displayed a significant increase in the relative abundance of *Proteobacteria* compared with that in the naïve group, whereas the relative abundance of *Actinobacteria* was significantly decreased (*p* < 0.05) (Figure 3C). At the genus level, compared with the naïve group, the relative abundance of *Pseudomonas* in the model group significantly increased, whereas the relative abundance of *Bifidobacterium* was significantly decreased, that was reversed by MFXD (Figures 3D,E). These findings indicated that MFXD could attenuate lung microbiota dysbiosis in OVA-induced AR mice, resulting in the microbiota composition of AR mice close to that of the naïve group.

### 3.5. MFXD altered plasma metabolite in AR mice

**Figure 4:**
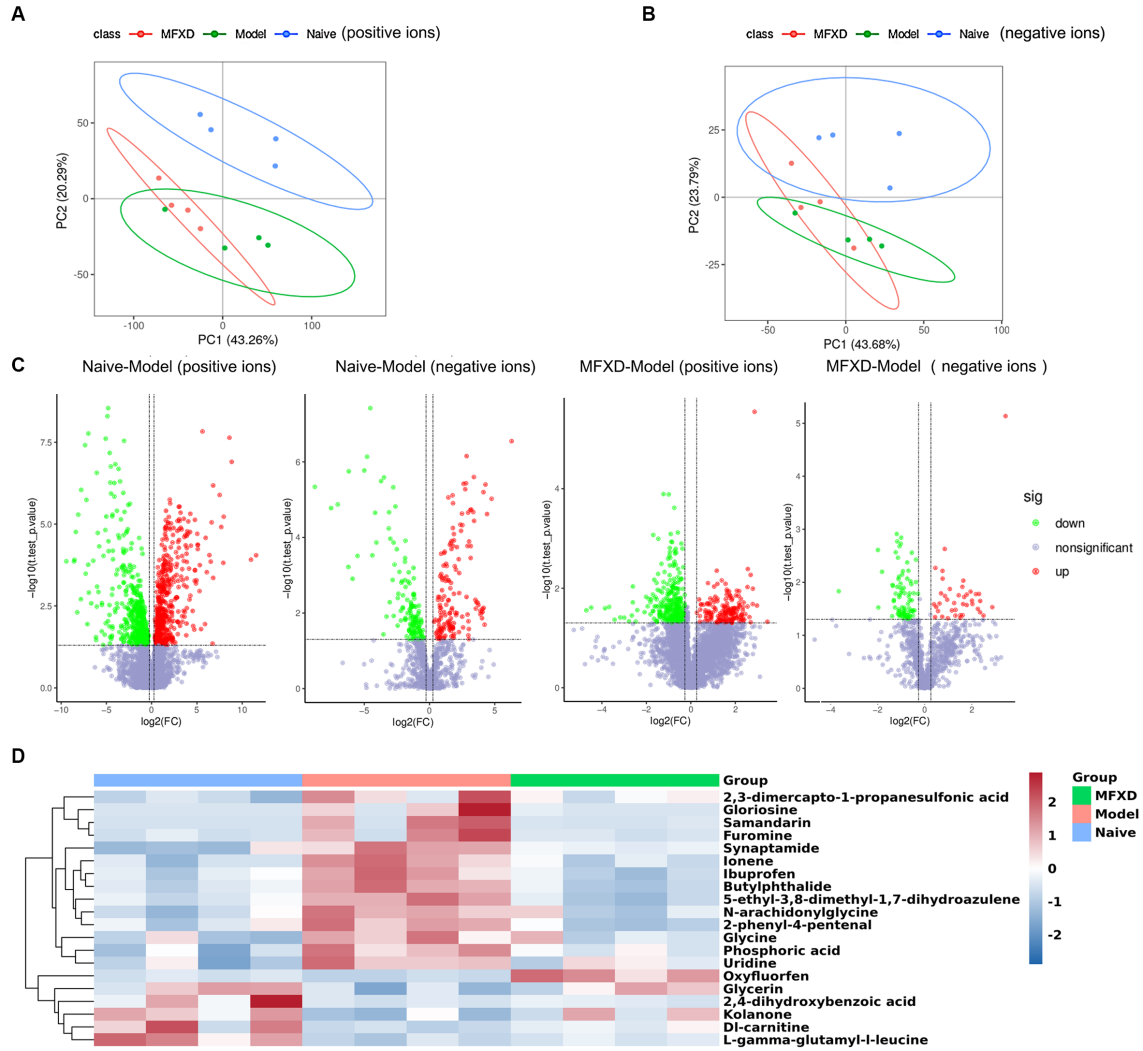
Mahuang Fuzi Xixin decoction (MFXD) altered plasma metabolite in AR mice. **(A)** Principal component analysis (PCA) in the positive ions and **(B)** negative ions. **(C)** Volcano plots. **(D)** the top 20 metabolites significantly regulated by MFXD.

LC-MS/MS was used to perform an untargeted metabolomic analysis to reveal the specific metabolites of AR mice and the protective effect of MFXD. In the positive ion mode, the PCA plots showed the samples in the AR model group were separated from those in the naïve group, and the samples in the MFXD group tended to approach those in the naïve group. This trend was more apparent in the negative ion mode (Figures 4A,B). A total of 974 differential metabolites were identified between naïve and AR model groups in the positive ion mode, of which 504 metabolites were up-regulated and 470 were down-regulated. There were 452 differential metabolites have been detected between the MFXD and AR model groups, of which 173 were up-regulated and 279 were down-regulated (Figure 4C). In the negative ion mode, 290 differential metabolites were identified between the naïve and model groups (125 up-regulated and 138 down-regulated), whereas 122 (40 up-regulated and 82 down-regulated) were identified between the MFXD and model groups (Figure 4C). OVA dramatically changed the metabolites in mice plasma compared to the naïve mice, while the altered metabolites were significantly reversed by MFXD (Supplementary File 1) and Figure 4D showed the top 20 metabolites significantly regulated by MFXD, including kolanone, L-gamma-glutamyl-l-leucine, 2,3-dimercapto-1-propanesulfonic acid, and 2,4-dihydroxybenzoic acid, etc.

**Figure 5:**
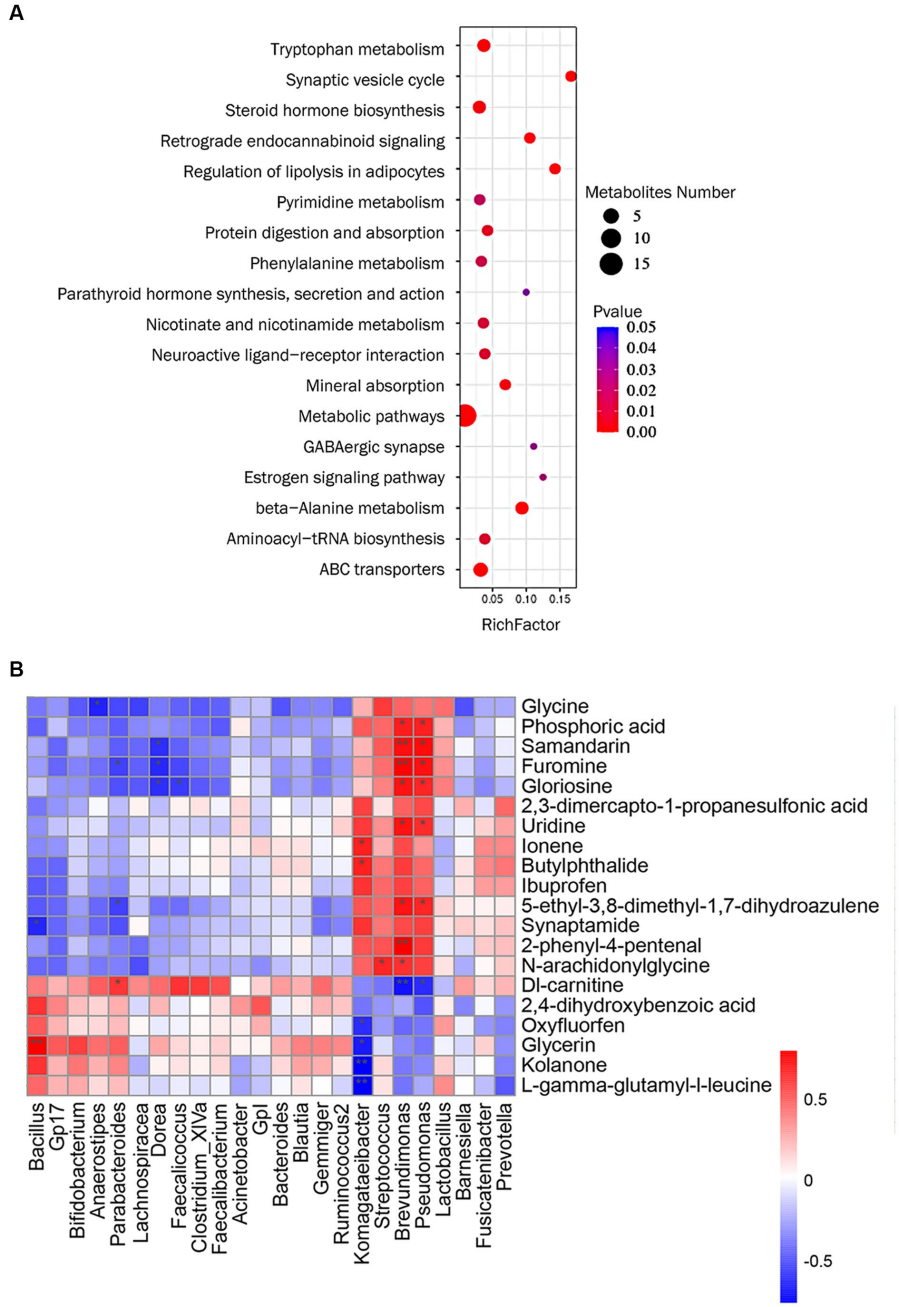
**(A)** KEGG pathway analysis of metabolites significantly regulated by Mahuang Fuzi Xixin decoction (MFXD). **(B)** Correlation analysis among metabolites and lung microbiota. ^*^p < 0.05, ^**^p < 0.01.

We performed KEGG pathway enrichment analysis on differential metabolites and the results showed that MFXD-regulated metabolites were mainly involved in beta-alanine metabolism, steroid hormone biosynthesis, tryptophan metabolism, etc. (Figure 5A).

### 3.6. Correlation analysis among the lung microbe and plasma metabolites

We used *Spearman*’s correlation analysis to measure the degree of association between lung microbes and plasma metabolites. The results showed that 17 plasma metabolites, including samandarin, Dl-carnitine, uridine, furomine, gloriosine, N-arachidoninglycine, 2-phenyl-4-pental were correlated with the alterations of 8 genus lung microbes (Figure 5B). For example, the relative abundance of *Pseudomonas* and *Brevundimonas* exhibited a positive correlation with uridine, gloriosine, furomine, phosphoric acid, 5-ethyl-3,8-dimethyl-1,7-dihydroazulene, and samandarin; and significant negative correlations with Dl-carnitine; The relative abundance of *Komagateibacter* is significantly negatively correlated with kolanone, L-gamma-glutamyl-l-leucine, glycerin, and oxyfluorfen, positively correlated with ionene and butylphthalide. These results indicate that there is a certain correlation between lung microbe and plasma metabolites, and MFXD can regulate the disorder of lung microbe and plasma metabolites in the treatment of AR.

### 3.7. Effects of MFXD-LP on the viability of HNEpCs

**Figure 6:**
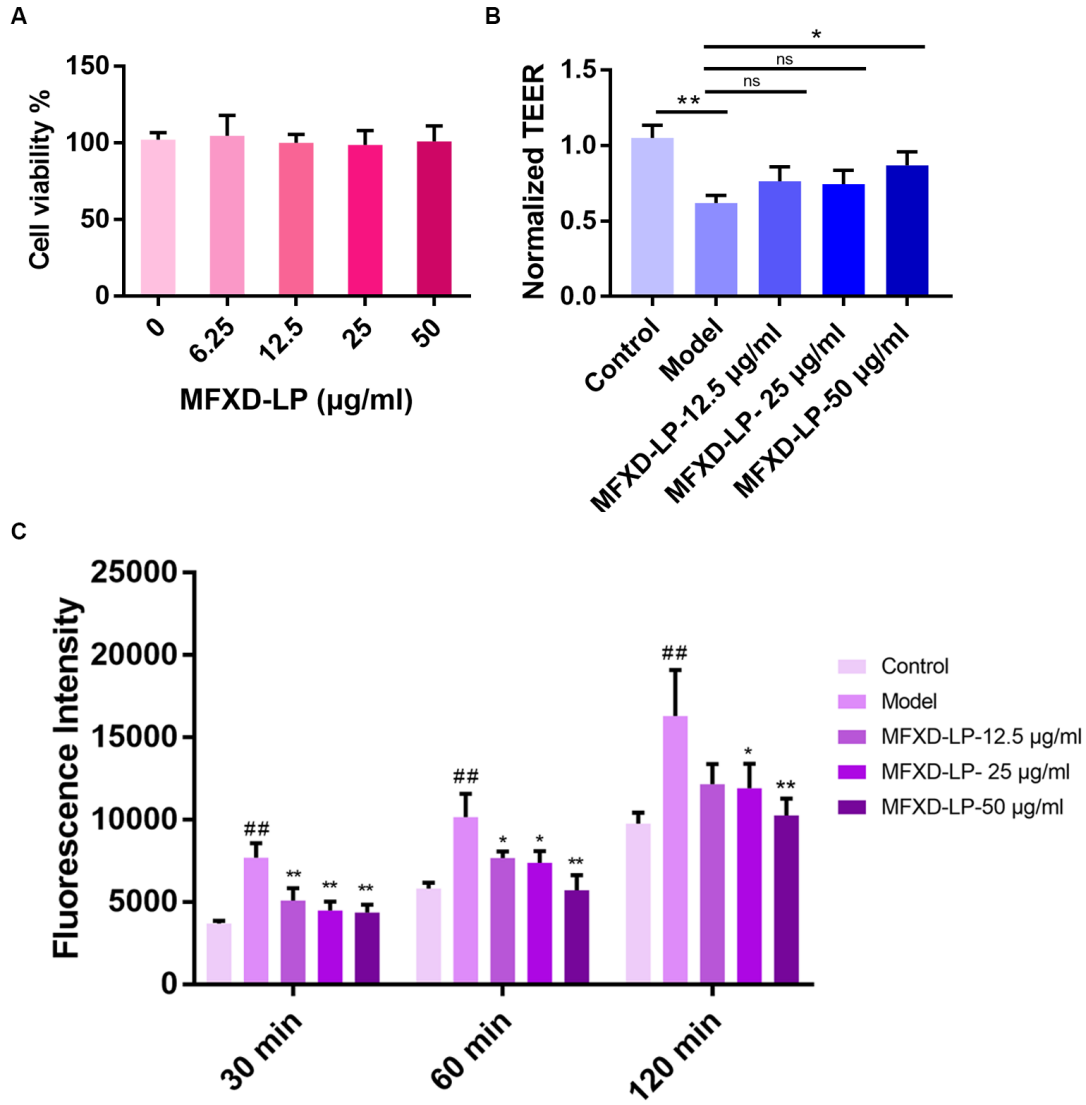
Effects of Mahuang Fuzi Xixin decoction lyophilized powder (MFXD-LP 6.25–50 μg/mL) on **(A)** cell viability; **(B)** trans epithelial electrical resistance (TEER); **(C)** FITC-FD4 permeability. ^*^p < 0.05, ^**^p < 0.01.

The effect of MFXD-LP on the viability of HNEpCs was evaluated by CCK-8 kits. 12.5, 25, and 50 μg/mL of MFXD-LP did not affect the viability of HNEpCs (Figure 6A).

### 3.8. MFXD improved the PAE-induced decrease in TEER and the increase in FITC-FD4 transmembrane permeability

We investigated whether MFXD has a protective effect on the PAE-induced decrease in the TEER of HNEpCs monolayers. As we expected, compared with that of the control group, PAE significantly (*p* < 0.01) induced the reduction of TEER and significantly (*p* < 0.01) increased paracellular permeability of HNEpCs, treatment of MFXD-LP could reverse it (Figures 6B,C).

### 3.9. MFXD restored PAE-induced tight junction proteins disruption

**Figure 7:**
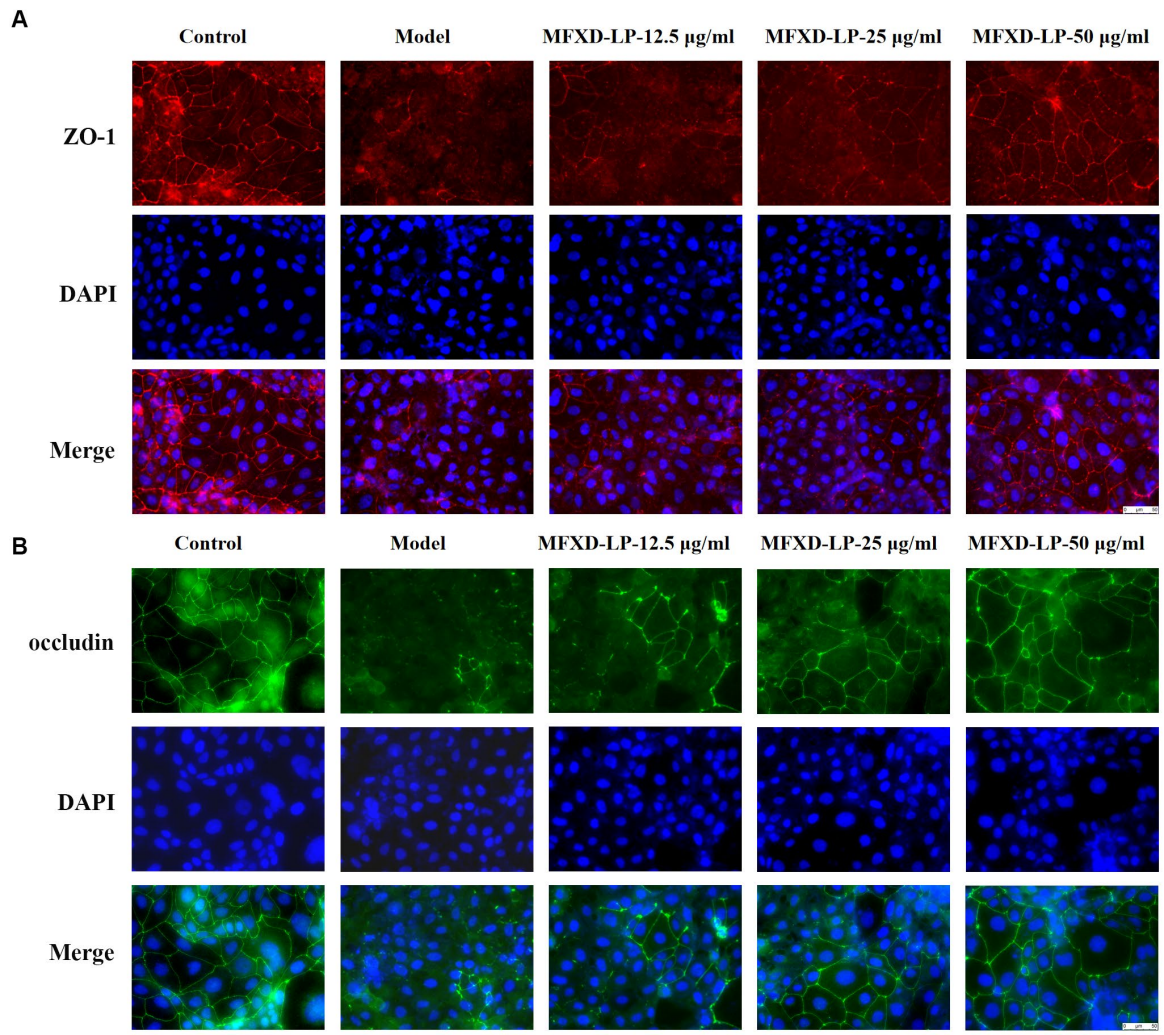
Mahuang Fuzi Xixin decoction (MFXD) restored PAE-induced tight junction proteins disruption **(A)** ZO-1 and **(B)** occludin.

Furthermore, immunofluorescence (IF) was used to assess the protective effect of MFXD on PAE-induced tight junction proteins disruption. Treatment with PAE downregulated the expression of ZO-1, claudin-1, and occludin, MFXD-LP (12.5, 25, or 50 μg/mL) markedly reversed it in a dose-dependent manner (Figure 7). These results indicate that MFXD has a protective effect on PAE-induced tight junction proteins disruption.

### 3.10. MFXD inhibited the growth of *Pseudomonas aeruginosa*

**Figure 8:**
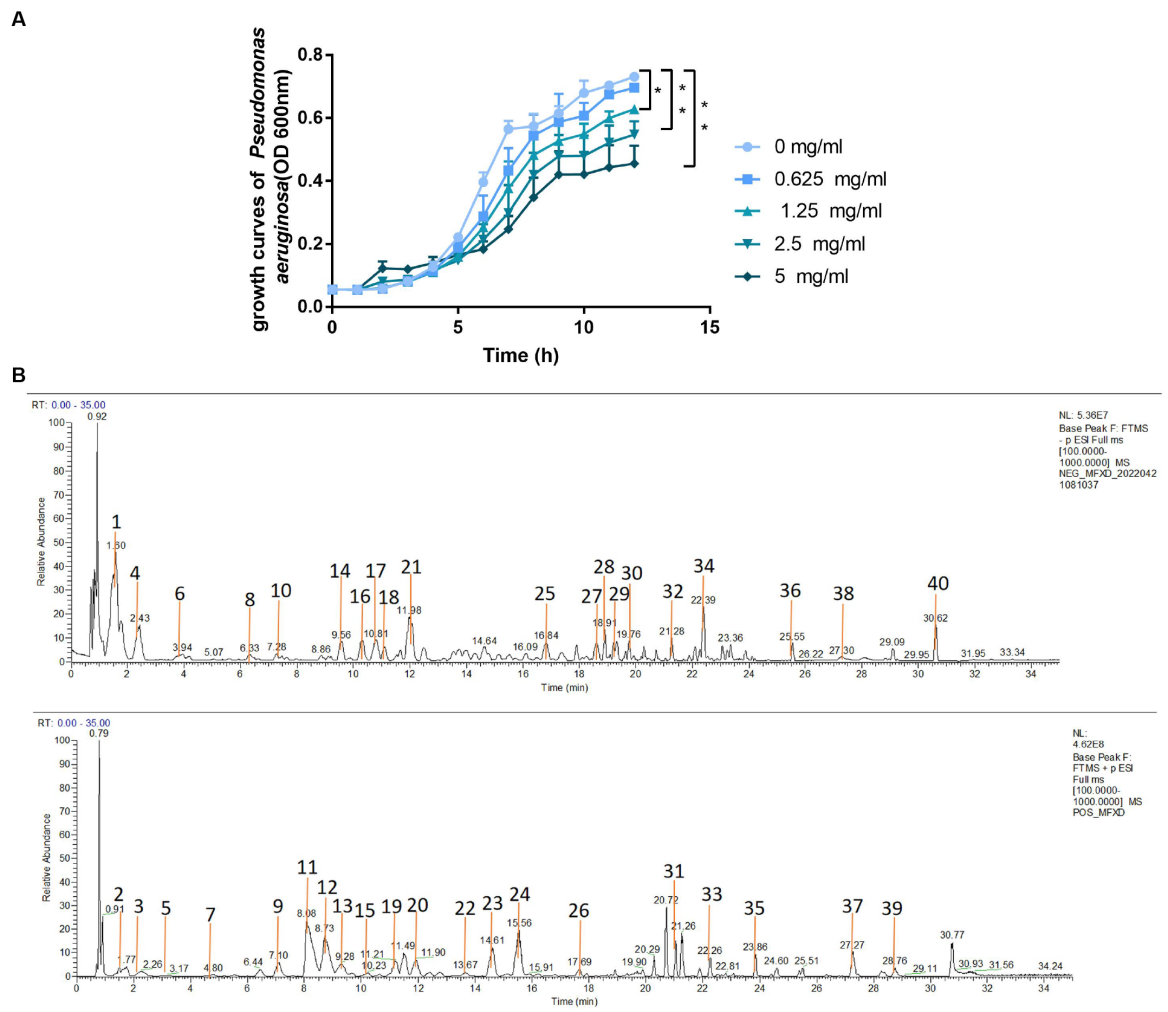
**(A)** Growth curve of *Pseudomonas aeruginosa* **(B)** the total ion current chromatograms of MFXD. ^*^p < 0.05, ^**^p < 0.01.

We evaluated the effect of MFXD on the growth of *P. aeruginosa*. MFXD-LP (1.25, 2.5, and 5 mg/mL) markedly inhibited the growth rate of *P. aeruginosa* (Figure 8A). This indicates that MFXD not only repairs the damage to tight junction proteins caused by PAE but also directly inhibits the growth of *P. aeruginosa*.

### 3.11. Chemical characterization of MFXD

To identify the active components in MFXD, the prepared MFXD sample was analyzed by LC-MS/MS both in positive ESI ± and negative ESI− mode. A total of 40 components were identified (Figure 8B), inclucding ephedrine, pseudoephedrine, etc. The details of identification information are provided in the Supplementary File 2.

## 4. Discussion

AR is a common inflammatory respiratory disorder closely associated with asthma. Gut microbes are considered to be closely related to the pathogenesis of AR. Although the lungs are connected to the upper respiratory tract, which is rich in microorganisms, research on the relationship between lung microbes and airway disease is very limited. MFXD, a TCM formula widely used in the treatment of AR, has been proven to regulate intestinal flora in AR rats (Li and Li, 2014; Liang et al., 2020). However, its effect on lung microbiota has not been reported yet. In this study, MFXD protected mice against OVA-induced AR, significantly decreased itching and sneezing incidences, and down-regulated histamine and IgE expression. Simultaneously, MFXD ameliorated nasal and bronchial mucosal damage, and restored nasal and bronchial mucosal barrier function. We demonstrated for the first time that lung microbiota plays a vital role in AR, MFXD altered the lung microbiota dysbiosis and abnormalities of plasma metabolites in AR mice. We found that the dominant bacteria in the lungs of AR mice could damage the airway barrier, and MFXD could reverse it.

Microbiota in the lung largely reflects the bacterial colony that enters the lung through respiration in the upper respiratory tract. It has been demonstrated that there is a significant correlation observed in the bacterial culture between the upper and lower respiratory tracts (Ramakrishnan et al., 2013). Emerging reports suggest that microbial dysbiosis of lung microbiota is involved in lung cancer, asthma, chronic obstructive pulmonary disease, etc. (Yagi et al., 2021). Microbiota can affect the immune response and microbial composition, by affecting lymphocytes in the intestinal and respiratory tract and by lymphocyte migration (Budden et al., 2017). Specific microbiota can also induce Th cell polarization and cytotoxic CD8 T cell activity (Shim et al., 2023). During dysbiosis, epithelial damage may cause bacterial metabolites to enter the circulatory system. In this study, the results of 16S rDNA amplicon sequencing showed that the AR mice had a higher relative abundance of *Proteobacteria* and a lower abundance of *Actinobacteria* than the naïve and MFXD groups. Similar to our results, Wang et al. (2022) revealed that in patients with asthma, the abundance of *Actinobacteria* may be negatively associated with neutrophils and participate in airway inflammation. *Proteobacteria* was also increased in patients with asthma, which may correlate with bronchial hyperresponsiveness. At the genus level, a higher abundance of *Pseudomonas* and a lower abundance of *Bifidobacterium* were found in AR mice compared with the naïve and MFXD groups. *Bifidobacterium* is reported to be associated with immunomodulatory activities, and intranasal administration of *Bifidobacterium longum* protected mice from influenza-induced lung inflammation and injury (Groeger et al., 2020). Gan et al. (2021) reported that in the nasal of AR patients, the abundance of *Pseudomonas* is higher than that in the control. *P aeruginosa* infections are reported to be implicated in asthma, atrophic rhinitis, and chronic rhinosinusitis (Tuli et al., 2021). An increase in its abundance may further cause an immune response and barrier disruption thus, promoting the AR process.

In the metabolomics study, we identified 25 metabolites that were elevated in the AR group and downregulated by MFXD, including uridine, L-gamma-glutamyl-L-leucine glycine, gamma-L-glutamyl-L-tyrosine, N-arachidoninglycine, 2-phenyl-4-pental, 2,3-dimercapto-1-propanesulfonic acid, etc. In allergic asthma, uridine can be converted into uridine diphosphate glucose (UDP-G), which can activate the purinergic receptor P2Y14R on eosinophils, causing their migration to the lungs and amplifying allergen-induced eosinophil aggregation (Foster et al., 2021). L-gamma-glutamyl-L-leucine is a key intermediate in the γ-glutamyl cycle, and has significant anti-inflammatory effects (Chen et al., 2019). Other metabolites are also related to allergic disease and microbial homeostasis, for example, 2,3-dimercapto-1-propanesulfonic acid (DMPS) was reported to induce allergic skin reactions in the patients (Erden et al., 2019). 2,4-dihydroxybenzoic acid (2,4-DHB), a derivative of benzoic acid, has been reported to possess strong antimicrobial properties against *P. aeruginosa* (Kalinowska et al., 2021). These metabolites involving tryptophan metabolism and pyrimidine metabolism, β-alanine metabolism and other pathways.

Microbiota plays an important role in systemic metabolism, affecting nearly half of the blood metabolites (Visconti et al., 2019). Our previous study revealed that MFXD altered gut microbiota dysbiosis and improved the increase in short-chain fatty acids (SCFA) content to ameliorate AR (Liang et al., 2020). In this study, correlation analysis showed that *Brevundimonas, Pseudomonas,* and *Komagataeibacter* in the lung are the most closely related genera to differential metabolites, showing significant interrelationship. The relative abundance of *Pseudomonas* is significantly positive correlated with uridine, gloriosine, furomine, phosphoric acid, 5-ethyl-3,8-dimethyl-1,7-dihydroazulene and samandarin, they all decreased after MFXD treatment; the relative abundance of *Pseudomonas* is negatively correlated with DL-carnitine, which is increased in MFXD group, is reported as an inhibitor of *P. aeruginosa* biofilm development (Wenderska et al., 2011). These results indicate that microbiota and metabolites are interrelated, and MFXD can play a therapeutic role in AR by influencing metabolites and microbiota.

The nasal epithelial barrier is the first line for the body to resist risk factors. Allergens and inflammatory factors from multiple sources can cause damage to the airway barrier, restoring the nasal epithelial barrier is considered a therapeutic approach to AR (Zhang et al., 2023). Immunohistochemistry showed that the expression of tight junction proteins in the lung and nasal mucosa of AR mice was decreased, which was reversed by oral administration of MFXD. Microbiota is also involved in preserving the integrity of the epithelial barrier. Probiotics can compete with pathogenic bacteria for nutrition by adhering to the mucosa, thereby inhibiting the growth of pathogenic bacteria (Rawal and Ali, 2023). We found a decrease in *Bifidobacterium* in AR mice and an increase after MFXD treatment. Studies have shown that *Bifidobacterium* improves intestinal barrier function, and given the similarities between the intestinal and airway epithelial barriers (Krumbeck et al., 2018), it is likely that *Bifidobacterium* in the airway can also confer a protective effect on the airway barrier.

*P. aeruginosa* is a ubiquitous opportunistic pathogen, hypersecretion of mucus in the nasal and lungs caused by rhinitis or asthma provides an abundant medium for *P. aeruginosa* colonization, and its exoproteins have been shown to cause mucosal barrier disruption (Hansen et al., 2012; Tuli et al., 2021). In this study, the relative abundance of *Pseudomonas* in the AR group was significantly increased. Therefore, we inferred that the increase in the relative abundance of *Pseudomonas* in AR mice may lead to tight junction protein disruption, which affects the progression of AR. The permeability of FD4 and TEER in the cell model also showed that *P. aeruginosa* increased the permeability and decreased the TEER of monolayer cells, it has been reversed after MFXD treatment. Moreover, MFXD-LP not only protected tight junctions but also significantly inhibited the growth of *P. aeruginosa* at a concentration of 1.25 mg/mL. These data suggest that MFXD can inhibit the growth of *P. aeruginosa* and regulate airway barrier damage caused by it. This may be one of its mechanisms in the treatment of AR. However, up to now, the understanding of lung microbes is still in its infancy, and the presence of microbiota in the lungs of healthy individuals may be short-lived, but the increase in dominant bacteria in patients with AR may cause pulmonary microorganisms to persist and affect the progression of the disease. A more in-depth study should be carried out in the future by using sterile mice, antibiotic intervention, flora transplantation, etc.

## 5. Conclusion

In conclusion, this is the first study to investigate the changes in the lung microbiota and the correlation analysis of microbiota in the lung and blood metabolites in the AR model. We confirmed that lung microbiota plays a vital role in AR, and the excessive proliferation of lung pathogenic microbiota may further promote the development of AR. MFXD reduced damage to the epithelial barrier of the lung and nasal mucosa by regulating the dysbiosis of lung microbes and plasma metabolites. Our research provides a reference for the effect of lung microbiota on AR and provides a new strategy for the treatment of AR.

## Data availability statement

The datasets presented in this study can be found in online repositories. The names of the repository/repositories and accession number(s) can be found at: https://www.ncbi.nlm.nih.gov/, PRJNA952406.

## Ethics statement

All animal experiments have passed the resolution of the Animal Ethics Committee of Southern Medical University (Resolution No. SMUL2019087; Date of Resolution 2019.5.10).

## Author contributions

XW wrote the paper draft and mainly conducted this study. MD, XL, and BZ provided help on animal handling. XT provided funding to support the study. ZZ supervised the experimentators. All authors contributed to the article and approved the submitted version.

## Funding

This work was supported by the Natural Science Foundation of Guangdong Province (grant number 2022A1515011649), and the Middle-aged and Young Teachers’ Basic Ability Promotion Project of Guangxi (grant number 2021KY0513).

## Conflict of interest

The authors declare that the research was conducted in the absence of any commercial or financial relationships that could be construed as a potential conflict of interest.

## Publisher’s note

All claims expressed in this article are solely those of the authors and do not necessarily represent those of their affiliated organizations, or those of the publisher, the editors and the reviewers. Any product that may be evaluated in this article, or claim that may be made by its manufacturer, is not guaranteed or endorsed by the publisher.

## Footnotes

^1^http://metlin.scripps.edu

^2^http://www.hmdb.ca/

^3^https://pathview.uncc.edu/home

## Glossary

AR: Allergic rhinitis
ELISA: Enzyme-linked immunosorbent assay
H&E: Hematoxylin and eosin
HNEpCs: Human nasal epithelial cell lines
HRMS: High-resolution mass spectrometry
IgE: Immunoglobulin E
IHC: Immunohistochemistry
IS: Internal standards
MFXD: Mahuang Fuzi Xixin decoction
MS: Mass spectrometry
OVA: Ovalbumin
PAE: Pseudomonas aeruginosa exoprotein
PCR: Polymerase chain reaction
PLS-DA: Partial least squares discriminant analysis
PVDF: Polyvinylidene difluoride
QC: Quality control
SPF: Specific pathogen-free
TEER: Transepithelial electrical resistance
TCM: Traditional Chinese medicine
UPLC: Ultra high-performance liquid-phase chromatography
ZO-1: Zonula occludens-1
