# Supplementary material for: Mahuang Fuzi Xixin decoction ameliorates allergic rhinitis and repairs the airway epithelial barrier by modulating the lung microbiota dysbiosis
Source: Front Microbiol. 2023 Aug 14;14:1206454. doi: 10.3389/fmicb.2023.1206454 (PMC10461068; doi:10.3389/fmicb.2023.1206454)
Supplement: Supplementary file 1 [file Table_1.doc]

Supplementary Material

# Mahuang Fuzi Xixin decoction ameliorates allergic rhinitis and repairs the airway epithelial barrier by modulating the lung microbiota dysbiosis

Xiaohan Wei, Mengze Ding, Xiao Liang, Baoping Zhang, Xiaomei Tan*, Zezhong Zheng*

*Correspondence:

Zezhong Zheng E-mail addresses: zezhong@scau.edu.cn

Xiaomei Tan E-mail addresses: tanxm_smu@163.com

# Supplementary File 1 :

**Supplementary Table 1 Metabolites significantly regulated by MFXD.**

| Group | q.value | Name | Formula | Molecular  Weight | RT | Mode | Label |
| --- | --- | --- | --- | --- | --- | --- | --- |
| MFXD:Model | 0.031 | Uridine | C_9_H_12_N_2_O_6_ | 244.0695 | 0.914 | pos | down |
| MFXD:Model | 0.0148 | Synaptamide | C_24_H_37_ NO_2_ | 371.2826 | 8.908 | pos | down |
| MFXD:Model | 0.0279 | Samandarin | C_19_H_31_NO_2_ | 305.2353 | 6.28 | pos | down |
| MFXD:Model | 0.0039 | Phosphoric acid | H_3_O_4_P | 97.9768 | 0.801 | pos | down |
| MFXD:Model | 0.0385 | N-arachidonylglycine | C_22_H_35_NO_3_ | 361.2617 | 8.948 | pos | down |
| MFXD:Model | 0.0224 | Ionene | C_13_H_18_ | 174.1408 | 8.963 | pos | down |
| MFXD:Model | 0.0397 | Ibuprofen | C_13_H_18_O_2_ | 206.1307 | 8.948 | pos | down |
| MFXD:Model | 0.0179 | Glycine | C_2_H_5_NO_2_ | 75.032 | 0.948 | pos | down |
| MFXD:Model | 0.0246 | Gloriosine | C_21_H_23_NO_6_ | 385.1535 | 9.12 | pos | down |
| MFXD:Model | 0.0393 | Furomine | C_20_H_32_N_2_O_4_ | 364.2364 | 6.802 | pos | down |
| MFXD:Model | 0.023 | Butylphthalide | C_12_H_14_O_2_ | 190.0993 | 8.96 | pos | down |
| MFXD:Model | 0.0378 | 5-ethyl-3,8-dimethyl-1,7-dihydroazulene | C_14_H_18_ | 186.1408 | 8.962 | pos | down |
| MFXD:Model | 0.0132 | 2-phenyl-4-pentenal | C_11_H_12_O | 160.0888 | 8.956 | pos | down |
| MFXD:Model | 0.0477 | 1,1,6-trimethyl-1,2-dihydronaphthalene | C_13_H_16_ | 172.1252 | 8.951 | pos | down |
| MFXD:Model | 0.0182 | (z)-hex-3-enyl benzoate | C_13_H_16_O_2_ | 204.115 | 8.958 | pos | down |
| MFXD:Model | 0.0291 | 18-acetoxy-1alpha,25-dihydroxyvitamin d3 | C_29_H_46_O_5_ | 474.3348 | 7.776 | pos | up |
| MFXD:Model | 0.0354 | Dl-carnitine | C_7_H_15_NO_3_ | 161.1051 | 0.726 | pos | up |
| MFXD:Model | 0.0193 | Gamma-l-glutamyl-l-tyrosine | C_14_H_18_N_2_O_6_ | 310.1165 | 2.809 | pos | up |
| MFXD:Model | 0.0438 | Glycerin | C_3_H_8_O_3_ | 92.0473 | 0.788 | pos | up |
| MFXD:Model | 0.0112 | Kolanone | C_33_H_42_O_4_ | 502.3074 | 8.83 | pos | up |
| MFXD:Model | 0.0186 | L-gamma-glutamyl-l-leucine | C_11_H_20_N_2_O_5_ | 260.1372 | 3.829 | pos | up |
| MFXD:Model | 0.0076 | Oxyfluorfen | C_15_H_11_ClF_3_NO_4_ | 361.0329 | 8.294 | pos | up |
| MFXD:Model | 0.0365 | 4-undecylbenzenesulfonic acid | C_17_H_28_O_3_S | 312.1751 | 9.977 | neg | down |
| MFXD:Model | 0.0451 | 2,3-dimercapto-1-propanesulfonic acid | C_3_H_8_O_3_S_3_ | 187.9638 | 1.059 | neg | down |
| MFXD:Model | 0.0432 | 2,4-dihydroxybenzoic acid | C_7_H_6_O_4_ | 154.0265 | 3.502 | neg | up |
