# Supplementary material for: Mahuang Fuzi Xixin decoction ameliorates allergic rhinitis and repairs the airway epithelial barrier by modulating the lung microbiota dysbiosis
Source: Front Microbiol. 2023 Aug 14;14:1206454. doi: 10.3389/fmicb.2023.1206454 (PMC10461068; doi:10.3389/fmicb.2023.1206454)
Supplement: Supplementary file 2 [file Table_2.doc]

Supplementary Material

# Mahuang Fuzi Xixin decoction ameliorates allergic rhinitis and repairs the airway epithelial barrier by modulating the lung microbiota dysbiosis

Xiaohan Wei, Mengze Ding, Xiao Liang, Baoping Zhang, Xiaomei Tan*, Zezhong Zheng*

*Correspondence:

Zezhong Zheng E-mail addresses: zezhong@scau.edu.cn

Xiaomei Tan E-mail addresses: tanxm_smu@163.com

# Supplementary File 2 :

**Supplementary Table 2 Bioactive compounds in MFXD.**

| NO.1 | Name | Formula | Molecular Weight | RT [min] | Area (Max.) |
| --- | --- | --- | --- | --- | --- |
| 1 | Citric acid | C_6_H_8_O_7_ | 192.02634 | 1.584 | 356542571.7 |
| 2 | Dehydroascorbic acid | C_6_H_6_O_6_ | 174.01593 | 1.752 | 130542205.4 |
| 3 | L-Isoleucine L | C_6_H_13_NO_2_ | 131.0941 | 2.263 | 25052290.67 |
| 4 | Itaconic acid | C_5_H_6_O_4_ | 130.02598 | 2.404 | 100808823.3 |
| 5 | Hordenine | C_10_H_15_NO | 165.11479 | 3.163 | 83261453.88 |
| 6 | Synephrine | C_9_H_13_NO_2_ | 167.09403 | 3.907 | 6326747.887 |
| 7 | L-Phenylalanine L | C_9_H_11_NO_2_ | 165.07846 | 4.788 | 55194380.26 |
| 8 | Epigallocatechin | C_15_H_14_O_7_ | 306.07329 | 6.325 | 27896537.32 |
| 9 | Cathine | C_9_H_13_NO | 151.09923 | 6.463 | 220977538.9 |
| 10 | 4-Methoxysalicylic acid | C_8_H_8_O_4_ | 168.04159 | 7.344 | 1013519.404 |
| 11 | Ephedrine | C_10_H_15_NO | 165.11482 | 8.097 | 3025542531 |
| 12 | Pseudoephedrine | C_10_H_15_NO | 165.11483 | 8.735 | 2641925938 |
| 13 | Methylephedrine | C_11_H_17_NO | 179.13045 | 9.305 | 597857210.4 |
| 14 | Piscidic acid | C_11_H_12_O_7_ | 256.05763 | 9.567 | 75777044.33 |
| 15 | Higenamine | C_16_H_17_NO_3_ | 271.12011 | 10.203 | 12365110.03 |
| 16 | 4-Methoxyphenylacetic acid | C_9_H_10_O_3_ | 166.06221 | 10.383 | 773150.3397 |
| 17 | Catechin hydrate | C_15_H_14_O_6_ | 290.07833 | 10.81 | 100875589.6 |
| 18 | Ferulic acid | C_10_H_10_O_4_ | 194.05732 | 11.011 | 8299846.451 |
| 19 | 4-Nitrophenyl palmitate | C_22_H_35_NO_4_ | 377.25578 | 11.215 | 478626427.6 |
| 20 | Bullatine G | C_22_H_31_NO_3_ | 357.22954 | 11.924 | 577445501 |
| 21 | Caffeic acid | C_9_H_8_O_4_ | 180.04161 | 12.014 | 187118554.5 |
| 22 | Raspberryketone glucoside | C_16_H_22_O_7_ | 326.13572 | 13.672 | 163041934.1 |
| 23 | Fuziline | C_24_H_39_NO_7_ | 453.27223 | 14.6 | 893915354.6 |
| 24 | Neoline/Bullatine B | C_24_H_39_NO_6_ | 437.27684 | 15.525 | 1460433058 |
| 25 | 1,6-Bismaleimidoethane | C_14_H_16_N_2_O_4_ | 276.1105 | 16.831 | 697715.2331 |
| 26 | Pyridoxine dicaprylate | C_24_H_39_NO_5_ | 421.28209 | 17.672 | 196238631.1 |
| 27 | p-Hydroxybenzaldehyde | C_7_H_6_O_2_ | 122.03649 | 18.631 | 1037424.485 |
| 28 | Kaempferitrin | C_27_H_30_O_14_ | 578.16293 | 18.916 | 95459215.53 |
| 29 | Methyl vanillate | C_9_H_10_O_4_ | 182.05714 | 19.389 | 661108.8972 |
| 30 | Hyperoside | C_21_H_20_O_12_ | 464.0947 | 19.776 | 53692481.69 |
| 31 | Sinapic acid | C_11_H_12_O_5_ | 224.06781 | 20.948 | 2152112.794 |
| 32 | N,N-Dibutylethanolamine | C_10_H_23_NO | 173.17748 | 21.3 | 1910447.83 |
| 33 | Benzoylhypaconine | C_31_H_43_NO_9_ | 573.29338 | 22.262 | 281912706 |
| 34 | Geranyl glucoside | C_16_H_28_O_6_ | 316.18803 | 22.397 | 99355380.52 |
| 35 | Hypaconitine | C_33_H_45_NO_10_ | 615.30327 | 23.856 | 272116366.8 |
| 36 | 6-Gingerol | C_17_H_26_O_4_ | 294.18312 | 25.552 | 28488078.73 |
| 37 | n-Amylbenzene | C_11_H_16_ | 148.12462 | 27.269 | 3337513.646 |
| 38 | Pogostone | C_12_ H_16_O_4_ | 224.10461 | 27.296 | 269591.052 |
| 39 | α-Cyperone | C_15_ H_22_O | 218.16643 | 28.798 | 1870535.394 |
| 40 | Octapinol | C_15_H_31_NO | 241.23992 | 30.628 | 133640.4764 |
